# Sex-Biased Gene Expression of *Mesobuthus martensii* Collected from Gansu Province, China, Reveals Their Different Therapeutic Potentials

**DOI:** 10.1155/2021/1967158

**Published:** 2021-08-19

**Authors:** Songyu Gao, Feng Wu, Xintong Chen, Ying Yang, Yina Zhu, Liang Xiao, Jing Shang, Xiaowei Bao, Yi Luo, Haihu Chen, Qing Liu

**Affiliations:** ^1^Faculty of Naval Medicine, Naval Medical University (Second Military Medical University), Shanghai 200433, China; ^2^College of Marine Science, Shanghai Ocean University, Shanghai 201306, China; ^3^College of Animal Science and Veterinary Medicine, Shanxi Agricultural University, Jinzhong, Shanxi 030801, China; ^4^School of Nursing, Naval Medical University (Second Military Medical University), Shanghai 200433, China; ^5^College of Food Science and Pharmacy, Xinjiang Agricultural University, Urumqi 830052, China; ^6^Central Medical District of Chinese PLA General Hospital, Beijing 100120, China; ^7^Department of Intervention, Changhai Hospital, Naval Medical University (Second Military Medical University), Shanghai, China

## Abstract

The scorpions, named *Mesobuthus martensii*, commonly called Quanxie (全蝎) in Chinese, have been widely used as one of the animal medicines for more than 1,000 years because of the strong toxicity of their venoms. Meanwhile, scorpions are sexually dimorphic in appearance, and many exhibit traits associated with sex-biased gene expression, including maternal care, mating competition, female mating choices, ecology, and even venom composition and lethality. This study aims to explore the differences in composition of the venom of scorpions of different sex using the method of transcriptomics. Whole *de novo* transcriptomes were performed on the samples of *M. martensii* captured from Gansu Province to identify their sex-biased gene expression. The conserved CO-1 sequences of the captured samples matched that of *M. martensii*. A total of 8,444 (35.15%), 7,636 (31.78%), 8,510 (35.42%), 7,840 (32.63%), 9,980 (41.54%), and 11,829 (49.23%) unigenes were annotated with GO, KEGG, Pfam, Swissprot, eggNOG, and NR databases. Moreover, a total of 43 metalloproteases, 40 potassium channel toxins, 24 phospholipases, 12 defensins, 10 peroxiredoxins, 9 cysteine proteinase inhibitors, 7 serine protease inhibitors, 6 sodium channel toxins, 2 NDBPs, 1 calcium channel toxin, 1 waprin-like peptide, 1 antibacterial peptide, 1 antimicrobial peptide, and 1 anticoagulant peptide were screened out. With the fold change of 2 and 0.5, *p* value < 0.01, and q value < 0.05 as thresholds, a total of 41 out of 157 (26.11%) toxin-related unigenes had significant differential expression, and this ratio was much higher than the ratio of differentially expressed unigenes out of all annotated ones (8.84%). Of these differentially expressed toxins, 28 were upregulated and occupied the majority, up to 68.30%. The female scorpions showed more upregulated unigenes that annotated with toxins and had the potential to be used as more effective therapeutic drugs. In addition, this method of omics can be further used as a useful way to identify the difference between female and male toxic animals.

## 1. Introduction

Variation is very common and important among different individuals of the same species, according to Darwinian evolution theory. There are differences between males and females in both humans and animals in the aspect of sex-specific reproductive tissues and also of size, shape, color, and behavior [1, 2], which are all sexually dimorphic traits. Sex-biased genes are called sex-specific genes if they are exclusively expressed in one sex, or sex-enriched genes if they are expressed at higher levels in one sex compared with the other [3]. In the process of growth and development, the abundance of sex-biased genes is constantly changing, which usually increases and peaks at a certain stage and then decreases in individual cases [4]. In addition, the level of differential expressed genes can be affected by environmental factors, such as the diet quality of *Drosophila melanogaster* and individual's social status of turkeys [5, 6].

Belonging to class Arachnida and phylum Arthropoda, the ancient terrestrial animal scorpions, we collected, almost have the same morphology characters of the scorpions discovered in the fossils, which began to form 400 million years ago in the Silurian Period [7]. According to the common treatment strategy called “combating poison with poison” in traditional Chinese medicine, scorpions were used as one of the animal medicines more than 1,000 years ago because of their strong toxicity [8]. Like other animals in nature, scorpions are also sexually dimorphic in appearance and show many traits associated with sex-biased gene expression, including maternal care, mating competition, female mating choice, venom composition, and lethality [9, 10]. Male scorpions have smaller bodies with longer tail segments, whereas females are larger with shorter and more rounded tail segments. The males are often faster and more flexible in searching for mates, whereas females are more sedentary because they are frequently gravid and always carry the young on their backs, making long-distance travels more difficult. The differences in ecological niches between males and females have resulted in the fact that females are more apt to sting defensively than males.

In the book of Shennong's Classic of Materia Medica, written in the Han Dynasty and regarded as the earliest known work of traditional Chinese medicine, the ancients had found there were many differences in the Chinese traditional medicines produced in different regions, and this viewpoint had been proved using modern technologies [11]. The concept of genuine medicinal materials formally put forward in the book of Collected Essentials of Species of Materia Medica completed in the Song Dynasty showed that the medicinal materials were produced in an area with specific natural conditions and ecological environment. In the same light, the sex was also regarded as an important influential factor of many traditional Chinese medicines, such as Tubiechong (*Eupolyphaga sinensis* Walker), Hamayou (*Oviductus Ranae*), Lurong (*Cornu cervi pantotrichum*), and the like. [12, 13]. In this article, the technique of transcriptomics was used to evaluate the differential expression levels of the toxins in scorpion venoms, which can be used as the main component of medicines, in the hope of laying a foundation for the studies on how to choose the sex of medicinal animals and improve their therapeutic efficacy.

## 2. Materials and Methods

### 2.1. Sample Collection

A total of 80 scorpions were collected from Gansu Province, China, in the summer of 2020. Half of them were female, and the others were male. They were sent to the laboratory immediately after collected and kept in a foam box fed on *Tenebrio molitor*. Their poison glands were cut from their telsons and were quickly frozen in liquid nitrogen and kept in −80°C environment.

### 2.2. Chemicals and Reagents

The Trizol reagent was purchased from Invitrogen (CA, USA). The RNA 1000 Nano LabChip Kit was purchased from Agilent (CA, USA). The mRNASeq sample preparation kit was purchased from Illumina (San Diego, USA). All other chemicals were of the highest purity commercially available.

### 2.3. Total RNA Extraction

Total RNA was extracted by Trizol reagent (Invitrogen, CA, USA). The manufacturer's procedures were strictly followed. The quantity and purity of total RNA were analyzed by Bioanalyzer 2100 and RNA 1000 Nano LabChip Kit (Agilent, CA, USA) with RIN number >7.0. Poly(A) RNA was purified from total RNA (5ug) using poly-T oligo-attached magnetic beads in two rounds of purification. After purification, the mRNA was fragmented into small pieces by divalent cations in the elevated temperature. Then, the cleaved RNA fragments were reverse-transcribed to create the final cDNA library in accordance with the protocol for the mRNASeq sample preparation kit (Illumina, San Diego, USA). The average insert size for the paired-end libraries was 300 bp (±50 bp). Then, we performed the paired-end sequencing on an IlluminaHiseq4000 at the (LC Sciences, USA), following the vendor's recommended protocol.

### 2.4. *De novo* Assembly, Unigene Annotation, and Functional Classification

First, Cutadapt [14] and Perl scripts in house were used to remove the reads that contained adaptor contamination, low-quality bases, and undetermined bases. Then, sequence quality was verified by FastQC (http://www.bioinformatics.babraham.ac.uk/projects/fastqc/), including the Q20, Q30, and GC content of the clean data. All downstream analyses were based on clean data of high quality. *De novo* assembly of the transcriptome was performed with Trinity 2.4.0 [15]. Trinity groups were transcribed into clusters based on shared sequence content. Such a transcript cluster is very loosely referred to as a “gene”. The longest transcript in the cluster was chosen as the gene sequence (aka unigene).

All assembled unigenes were aligned against the nonredundant (Nr) protein database (http://www.ncbi.nlm.nih.gov/), Gene ontology (GO) (http://www.geneontology.org), SwissProt (http://www.expasy.ch/sprot/), Kyoto Encyclopedia of Genes and Genomes (KEGG) (http://www.genome.jp/kegg/), and eggNOG (http://eggnogdb.embl.de/) databases using DIAMOND [16] with a threshold of E-value < 0.00001.

### 2.5. Differentially Expressed Unigenes Analysis

Salmon [17] was used to perform the expression level for unigenes by calculating TPM [18]. The differentially expressed unigenes were selected with log2 (fold change) > 1 or log2 (fold change) <−1 and with statistical significance (*p* value < 0.01 and q value < 0.05) by *R* package edgeR [19]. Next, GO and KEGG enrichment analyses were again performed on the differentially expressed unigenes by Perl scripts in-house.

### 2.6. Bioinformation Analysis

According to the level-1 domains' biological process, cellular components, and molecular functions, the GO (http://geneontology.org/) terms were analyzed. All unigenes were analyzed in contrast with the Kyoto Encyclopedia of Genes and Genomes (KEGG) database for their signaling pathways. The toxins or venomous transcripts from the scorpion samples were then searched against a toxin-related subdatabase from SWISSPROT (https://www.uniprot.org/program/Toxins). The protein sequences were translated from RNA sequences by the ORFfinder online service (https://www.ncbi.nlm.nih.gov/orffinder). From NCBI (https://blast.ncbi.nlm.nih.gov/Blast.cgi), we collected the sequences used for multiple sequence alignments and performed the alignments with the software Bioedit (version: 7.2.5). Phylogenetic trees were constructed in MEGA7 by the neighbor-joining method with bootstrap replication set at 1000. The 3D modeling was carried out in the software Discovery studio 2016.

## 3. Results

### 3.1. Quality Control and Overview of Transcriptome

**Figure 1:**
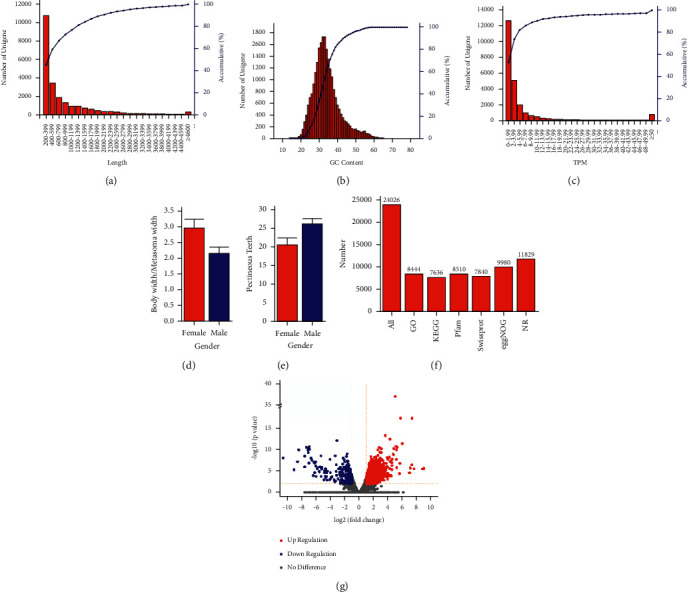
Quality control and overview of the transcriptome of male and female scorpions. (a) Length distribution of unigenes. (b) GC content distribution of unignens. (c) TPM distribution of unigenes. The horizontal coordinates of A, B, and C are the sequence length, GC content, and TPM of the unigenes, respectively. The left vertical coordinates of these histogram represent the number of unigenes, whereas the right vertical coordinates are the cumulative percentage of unigenes. (d) Body width/metasoma width of female and male samples. (e) Pectineous teeth number of female and male samples. (f) Annotation results with the database of GO, KEGG, Pfam, Swissprot, eggNOG, and NR, respectively. (g) Volcano plot of the differential expression unigenes. The red dots represent the upregulated unigenes, whereas the dark blue ones represent the downregulated unigenes.

The samples were collected from Gansu Province of China to explore the differences of the venoms between male and female scorpions by the method of transcriptome, which was regarded as an effective way to detect the differences in the gene level of the samples with different characters. The samples were sent to our laboratory lively with all body parts and with typical morphological characteristics of scorpions. They were kept in a foam box and fed on *Tenebrio molitor* temporarily. The collected scorpions were divided into two groups, male and female, according to the sex-biased characters, including the hardness of genital operculum and the width of sternum. Then, the body width and the metasoma width of the samples were measured and analyzed. The result of body width/metasoma width showed that female scorpions have higher value than the male ones (Figure 1(d)). Conversely, the male scorpions have more pectineous teeth in the pecten, which was consistent with the inherent characteristics of scorpions (Figure 1(e)). Eight groups of samples were tested, including 4 groups of females and 4 groups of males. A total of 47,842,140 (on average) raw reads were obtained with a GC ratio of more than 40% in all 8 groups of samples, after mRNA purification, cDNA synthesis, library construction, and Illumina sequencing. After the adapters and low-quality sequences were removed, whose quality value was less than 20, a total of 46,756,394 (97.7%, on average) valid reads were left (Table 1). The strategy of mixed *de novo* assembly of all the samples was adopted to get the unigenes. A total of 24,026 unigenes were obtained with a median length of 458 bp, and the total assembled bases number was 21,528,143 bp using the software Trinity. A total of 21,727 unigenes had a length from 200 to 2,199 bp and made up 90.43% of all unigenes. The three length distribution ranges with the largest proportion of unigenes were 200–399 bp, 400–599 bp, and 600–799 bp with a number of 10,771 (44.83%), 3,461 (14.40%), and 1,902 (7.92%), respectively (Figure 1(a)). The GC content of all the unigenes was 33.98%, and 95.79% unigenes had a GC content below 50% (Figure 1(b)). The TPM, which means the transcripts per kilobase of exon model per million mapped reads, was used as a normalized parameter to measure and compare the gene abundance among different samples (Figure 1(c)). With a BLASTx alignment e-value threshold of 10^−5^, we successfully annotated 8,444 (35.15%), 7,636 (31.78%), 8,510 (35.42%), 7,840 (32.63%), 9,980 (41.54%), and 11,829 (49.23%) unigenes with the GO, KEGG, Pfam, Swissprot, eggNOG, and NR databases (Figure 1(f)). From all the annotated unigenes, we screened 2,124 of them, which showed significant differential between the female and male scorpions and accounted for a percentage of 8.84%, while 2 and 0.5 were chosen as the threshold of the fold change and 0.01 for the *p* value and 0.05 for the q value. The majority of the differential expression unignens with a number of 1,722 (81.07%) were upregulated, whereas only 402 (18.93%) of them were downregulated (Figure 1(g)).

### 3.2. Species Identification

**Figure 2:**
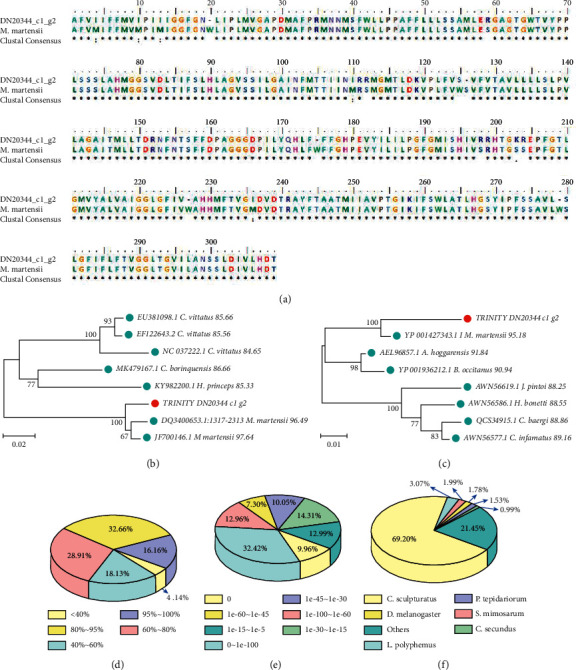
Species identification. (a) An amino acid sequences of COI from *M. martensii* aligned with the CO1 amino acids of DN20344 c1 g2 transformed from the DNA sequence by blastx. At the bottom of the columns, asterisks (^*∗*^) show conserved positions, and colons () show conserved substitutions and points. (b) Phylogenetic tree of mtDNA sequences of cytochrome c oxidase subunit I (MT-COI, CO1) constructed by the means of MEGA 7 with the Neighbor-Joining method. The sequencing DN20344 c1 g2 is marked with red dots and the arthropoda are marked with light blue dots. The identity values are indicated on the right of each species name. (c) Phylogenetic tree of the COI amino acid sequences of DN20344 c1 g2 and 7 other species constructed by means of MEGA 7 with the Neighbor-Joining method. The sequencing DN20344 c1 g2 is marked with red dots and the arthropoda are marked with light blue dots. The identity values are indicated to the right of each species name. (d) Similarity distribution for the annotation of NR database. (e) E-value distribution for the annotation of NR database. (f) Species distribution for the annotation of NR database.

The molecule mtDNA cytochrome c oxidase subunit I (MT-COI, COI), conventionally considered to be the signature sequence owing to its high conservation, was screened out from the contrasted transcriptome to make the species identification of the collected samples in molecular level [20]. With high identity and TPM in all the groups of samples, sequence DN20344 c1 g2 was chosen for further research among all the 6 COI sequences from annotation result to NR database. By the online tool of BLASTn (nucleotide to nucleotide), the most highly aligned sequence was sequence JF700146.1 from the species *Mesobuthus martensii* with an identity of 97.64%, followed by sequence DQ340065.1 also from the species *M. martensii* (Figure 2(b)). Moreover, by the online tool BLASTx (nucleotide to protein), the most aligned protein sequence was also from the species *M. martensii* with an identity of 95.18% (Figure 2(c)). By the software BioEdit, the amino acid sequence YP 001427343.1 was aligned with the CO1 amino acid sequences of DN20344 c1 g2 transformed from the DNA sequence by MEGA 7 (Figure 2(a)).

In conclusion, it is safe to say that all the collected scorpions belonged to the same species *M. martensii* after identified at the level of nucleotides and proteins. Compared with the NR database, a nonredundant protein sequence database collected by NCBI, the 80–95% similarity sequences were the most, accounting for 32.66% of all sequences, followed by the 60–80% similarity sequences with a percentage of 28.91% and the 40–60% similarity sequences with a percentage of 18.13% (Figure 2(d)). The e-value ranges of 0, 0∼1e-100, 1e-100∼1e-60, 1e-60∼1e-45, 1e-45∼1e-30, 1e-30∼1e-15, and 1e-15∼1e-5 represent 9.96%, 32.42%, 12.96%, 7.30%, 10.05%, 14.31%, and 12.99% of all sequences, respectively (Figure 2(e)). The top 4 aligned species were *Centruroides sculpturatus* (69.20%), *Limulus polyphemus* (3.07%), *Stegodyphus mimosarum* (1.99%), and *Drosophila melanogaster* (1.78%), and they all belong to Arthropoda as *M. martensii* (Figure 2(f)).

### 3.3. GO Analysis

**Figure 3:**
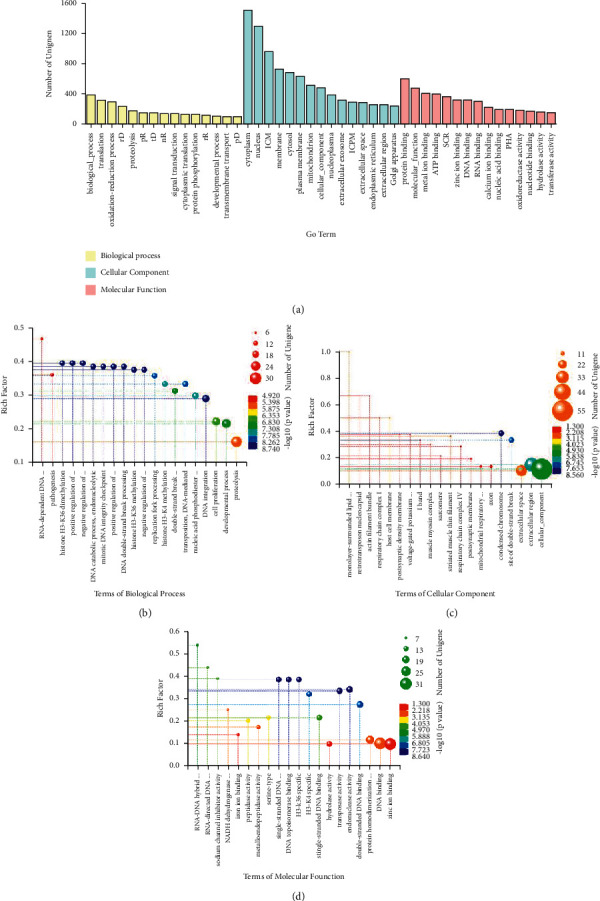
Gene Ontology (GO) annotation of unigenes. (a) The top fifteen terms of the three GO domains. (b) Bubble plots of the differential expression protein annotated in the biological process GO terms between female and male samples. (c) Bubble plots of the differential expression protein annotated in the cellular component GO terms between female and male samples. (d) Bubble plots of the differential expression protein annotated in the molecular function GO terms between female and male samples.

GO analyses of unigenes were conducted by BLAST2GO software with default parameters, and 8,444 unigenes (35.15%) were annotated to three primary GO domains, namely, biological process (BP), cellular component (CC), and molecular function (MF). The number of the annotated unigenes in the top three BP domain was 387, 312, and 292 accounting for the percentage of 1.78%, 1.44%, and 1.34% for the term of biological process, translation, and oxidation-reduction process. Cytoplasm, nucleus, and integral component of membrane occupied the top three terms with 1,509, 1,304, and 958 unigenes, which come up to the percentage of 6.95%, 6.00%, and 4.49% in CC domain. As for the domain of MF, the top three terms were protein binding, molecular function, and metal ion binding with 600 (2.76%), 479(2.20%), and 407 (1.87%) unigenes (Figure 3(a)). Subsequently, the differential expression unigenes were enriched in the GO terms, and the top twenty abundant terms were chosen for further analyses. Proteolysis showed the top differential expression unigenes in the domain of BP, reaching to 28, and also showed a rich factor of 0.16; however, the top rich factor in these terms was RNA-dependent DNA biosynthetic process with a rich factor of 0.47 and with differential expression unigenes of 7 (Figure 3(b)). For the CC domain, there were 55 differential expression unigenes in the term of cellular component, with a relatively low rich factor of 0.11, whereas the monolayer-surrounded lipid storage body term had a rich factor of 1 but the number of differential expressed unigenes in this term was only 2 (Figure 3(c)). The term of zinc ion binding was the most one in the MF domain with 31 unigenes and its rich factor was 0.10, while the maximum rich factor value was 0.54 in the term of RNA-DNA hybrid ribonuclease activity with 7 differential expression unigenes (Figure 3(d)).

### 3.4. KEGG Analysis

**Figure 4:**
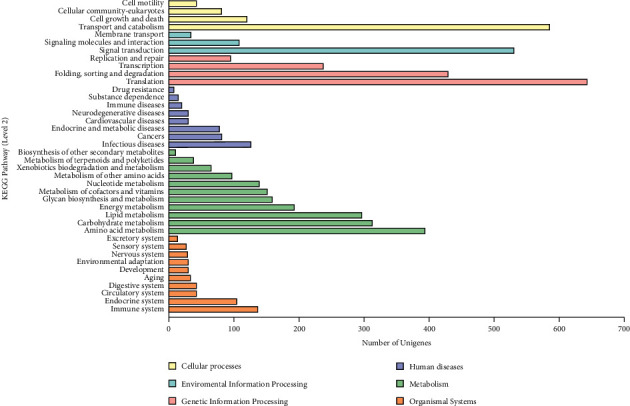
KEGG annotation of unigenes. The level 2 terms of KEEG are cellular process, environmental information processing, genetic information processing, human diseases, metabolism, and organismal systems.

A total of 7,636 unigenes (31.78%) were successfully annotated by KEGG databases, which was divided into six level-1 parts, namely, cellular process, environmental information processing, genetic information processing, human diseases, metabolism, and organismal systems. The top term was translation in genetic information processing with 644 unigenes, followed by transport and catabolism and then signaling molecules and interaction (Figure 4).

Cellular processes included transport and catabolism (586), cell growth and death (120), and cellular community-eukaryotes (81). Environmental information processing included signal transduction (530), signaling molecules and interaction (108), and membrane transport (34). Genetic information processing included translation (644), folding, sorting and degradation (430), and transcription (237). Human diseases included infectious diseases (127), cancers (82), and endocrine and metabolic diseases (78). Metabolism included amino acid metabolism (393), carbohydrate metabolism (313), and lipid metabolism (297). Organismal systems included immune system (136), endocrine system (103), and circulatory system (43) (Figure 4).

### 3.5. The Differential Expression of Potential Toxins

**Figure 5:**
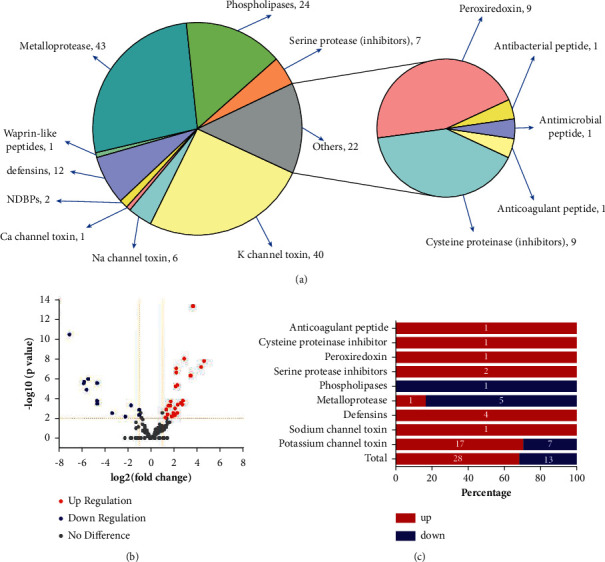
Classification of the scorpion venoms. (a) The classification of potential toxins screened from captured samples. (b) Volcano plots of differential expressed toxin-related unigenes. Each point in the map represents a differential unigene. The red points represent upregulated unigenes and the dark blue ones represent down-regulated unigenes. The abscissa represents the fold change, while the ordinate represents the statistical significance. (c) The number of up- or downregulated toxin-related unigenes. The abscissa represents the percentage, and the ordinate represents the kinds of the toxins. A Disintegrin and metalloproteinase.

By BLAST annotation of the transcriptome with the NR and SwissProt databases, a total of 158 toxin-related unigenes were screened and sorted according to their classification. The result showed that they included 43 metalloproteases, 40 potassium channel toxins, 24 phospholipases, 12 defensins, 10 peroxiredoxins, 9 cysteine proteinase inhibitors, 7 serine protease inhibitors, 6 sodium channel toxins, 2 NDBPs, 1 calcium channel toxin, 1 waprin-like peptides, 1 antibacterial peptide, 1 antimicrobial peptide, and 1 anticoagulant peptide (Figure 5(a), Table S1). Regarded the fold change of >2 or <0.5, *p* value < 0.01 and q value < 0.05 as thresholds, a total of 41 out of 158 (25.95%) had significant differential expression, and this ratio was much higher than that of differential expression unigenes in all annotated ones (8.84%). Of these differential expression toxins, 28 toxins were upregulated and occupied the majority, up to 68.30%. Besides, the ratio of differential expression unigenes varied in different kinds of toxins. As for the toxins we screened, all the 43 potassium channel toxins showed 24 differential expression unigenes and 17 of them were upregulated. We found 6 differential expression genes in 43 metalloproteases and only 1 in 24 phospholipases, indicating that the differential expression of toxin was mainly on potassium channel toxins. Besides, we found that 4 defensins, 2 serine protease inhibitors, 1 cysteine proteinase inhibitors, 1 sodium channel toxins, 1 peroxiredoxin, and 1 anticoagulant peptide were upregulated, and none of these toxins downregulated. Besides, the downregulated toxins were only distributed in potassium channel toxins, metalloproteases, and phospholipases (Figure 5(c)).

**Figure 6:**
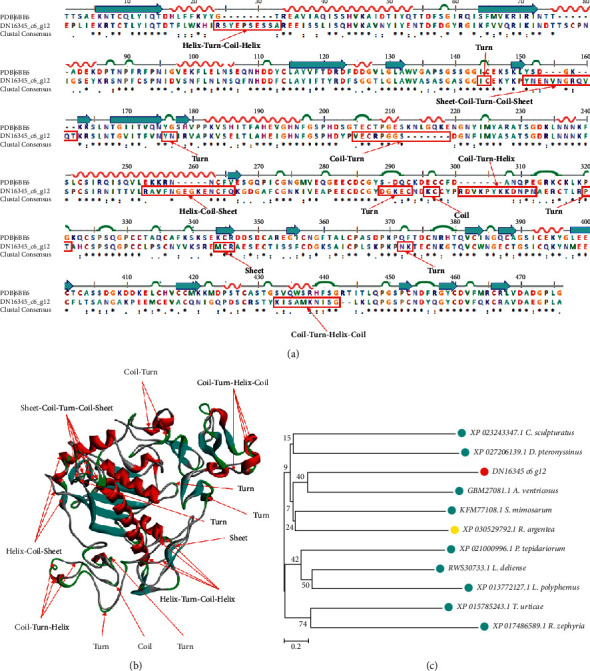
Sequence alignment, 3D modeling and phylogenetic analysis of a disintegrin and metalloproteinase. (a) A putative sequence Trinity_DN16345_c6_g12 was aligned with a model Adam (PDB ID: 6BE6). At the bottom of columns, asterisk (^*∗*^) shows conserved positions, colons (:) shows conserved substitutions, and points (.) shows nonconserved substitutions. Grey lines, green bends, blue-banded arrowheads, and red solenoids represent coils, turns, sheets, and helices respectively. Different fragments are framed by red lines. (b) 3D modeling was simulated by the template ADAM (PDB ID: 6BE6) in SWISS-MODEL and viewed in Discovery Studio 2016. The colors grey, green, blue, and red represent coils, turns, sheets, and helices, respectively. Different structures are indicated by red arrows. (c) Phylogenetic tree was constructed with sequence Trinity_DN16345_c6_g12, which is a putative ADAM and 10 other sequences from different species in MEGA 7, by the Neighbor-Joining method. The putative ADAM is marked in a red dot, arthropoda are marked in light blue dots, and Angiospermae are marked in a yellow dot.

As an abundant and diverse family of type-1 transmembrane metalloproteinases, a disintegrin and metalloproteinase (ADAM) are necessary for the developmental processes of numerous systems, including cardiovascular system, nervous system, immune system, gastrointestinal system, and the like [21–24]. ADAM endopeptidases can hydrolyze adhesion molecules, signaling receptors, cytokines, and growth factors. Besides, the dysfunction of ADAMs are associated with a lot of pathological states, such as Alzheimer's diseases, inflammation, osteoarthritis, kidney fibrosis, and even cancers [25–28]. In the transcriptome we constructed, the sequence Trinity_DN16345_c6_g12 was aligned with ADAM10 successfully. A model AFB1 (PDB ID: 6BE6) was used to perform the sequence alignment (Figure 6(a)) and 3D modeling (Figure 6(b)) of DN16345_c6_g12. The main structural variations contained a coil in 295KC298, five turns in 143IC146, 176YN178, 288DGKEC294, 319PT322, and 371NK374, a helix-turn-coil-helix in 23RSYEPSESSA34, a sheet-coil-turn-coil-sheet in 150YNENVNGRQVQT163, a coil-turn in 204VECRPGGS213, a helix-coil-sheet in 252RAVFNGEGKENCFQ267, a coil-turn-helix in 300RDVKPYKKDNPN313, a sheet in 343MCR347, and a coil-turn-helix-coil in 432KISAMKNISG443, respectively (Figure 6(a)). Phylogenetic analysis showed that the sequence DN16345_c6_g12 was evolutionally close to the sequence GBM27081.1 from *A.* ventricosus (Figure 6(c)).

### 3.6. Cathepsin L

**Figure 7:**
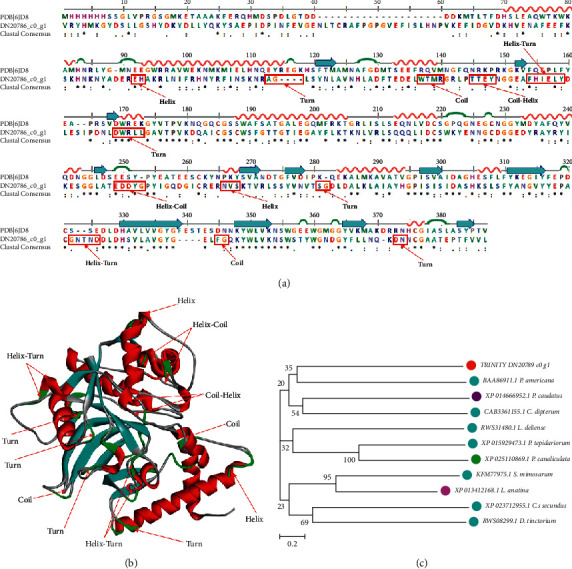
Sequence alignment, 3D modeling, and phylogenetic analysis of cathepsin L. (a) A putative sequenceTrinity_DN20786_c0_g1 was aligned with a model CTSL (PDB ID: 6JD8). At the bottom of columns, asterisk (^*∗*^) shows conserved positions, colons (:) shows conserved substitutions, and points (.) shows nonconserved substitutions. Grey lines, green bends, blue-banded arrowheads, and red solenoids represent coils, turns, sheets and helices respectively. Different fragments are framed by red lines. (b) 3D modeling was simulated by the template CTSL (PDB ID: 6JD8) in SWISS-MODEL and viewed in Discovery Studio 2016. The grey, green, blue, and red represent coils, turns, sheets and helices, respectively. Different structures are indicated with red arrows. (c) Phylogenetic tree was constructed with sequence Trinity_DN20786_c0_g1, which is a putative CTSL and 10 other sequences from different species, in MEGA 7, by the Neighbor-Joining method. The putative CTSL is marked in a red dot, arthropoda in light blue dots, priapulida in a purple dot, ollusca in a green dot, and brachiopoda in a pink dot.

As an important interfering factor in various biological processes, such as degradation and processing of protein, the cathepsins discovered in human body are sorted into 11 subtypes [29]. Because they can inhibit enzymatic activity in the process of invasion and metastasis of tumors, atherosclerosis, renal disease, and viral infection, Cathepsin *L* has become the potential therapeutic target in modern times. Besides, Cathepsin *L* is considered as a key intracellular lysosomal protease and provides a link between lysosomal dysfunction and frontotemporal lobar degeneration. Sequence Trinity_DN20786_c0_g1 was aligned with Cathepsin *L* (CTSL) successfully. A model CTSL (PDB ID: 6JD8) was used to perform the sequence alignment (Figure 7(a)) and 3D modeling (Figure 7(b)) of DN DN20786_c0_g1. The main structural variations contain 4 turns in 112AG115, 168DWRLL174, 280SG283, and 372DN375, 2 coils in 136WTMR141 and 344FG347, 2 helixes in 91EH94 and 365NVS269, 2 helix-turns in 153FHIELY160 and 321GNTND327, a coil-helix in 144TTEY149, a helix-coil in 248EDDYG254, respectively (Figure 7(a)). Phylogenetic analysis shows that the sequence DN20786_c0_g1 was evolutionally close to the sequence BAA86911.1 from *P. americana* (Figure 7(c)).

### 3.7. Peroxiredoxin IV;

**Figure 8:**
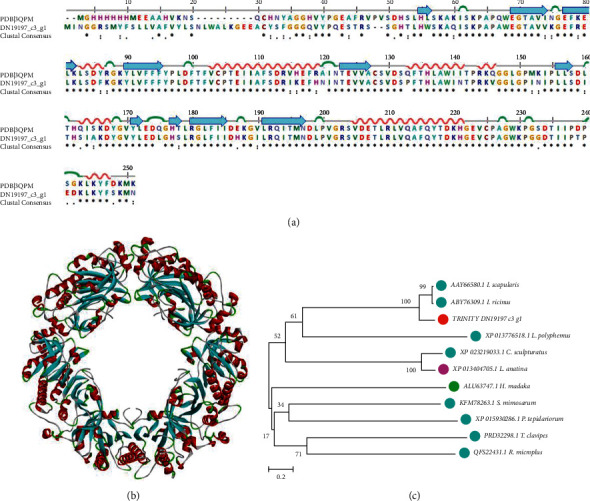
Sequence alignment, 3D modeling, and phylogenetic analysis of peroxiredoxin IV;. (a) A putative sequence sequence Trinity_DN19197_c3_g1 is aligned with a model PrxIV (PDB ID: 3QPM). At the bottom of columns, asterisk (^*∗*^) represent conserved positions, colons (:) represent conserved substitutions and points (.) represent nonconserved substitutions. Grey lines, green bends, blue-banded arrowheads, and red solenoids represent coils, turns, sheets, and helices, respectively. Different fragments are framed by red lines. (b) 3D modeling was simulated by the template PrxIV (PDB ID: 3QPM) in SWISS-MODEL and viewed in Discovery Studio 2016. The grey, green, blue, and red represent coils, turns, sheets, and helices, respectively. Different structures are indicated with red arrows. (c) Phylogenetic tree was constructed with sequence Trinity_DN19197_c3_g1, which is a putative PrxIV and 10 other sequences from different species, in MEGA 7, by the Neighbor-Joining method. The putative PrxIV is marked in a red dot, arthropoda in light blue dots, brachiopoda in a pink dot, and mollusca in a green dot.

Belonging to peroxidases, which can reduce the peroxides, peroxiredoxin is a abundant and highly conserved family [30]. Peroxiredoxin IV (PrxIV), one of this family, is the only known secretory form in this family. Besides, peroxiredoxin IV; is considered to have the ability to fight against diabetes mellitus, atherosclerosis, insulin resistance, and nonalcoholic fatty liver diseases by suppressing oxidative damage, inflammatory cytokines, and apoptotic activities [31–33]. In the transcriptome we constructed, the sequence Trinity_DN19197_c3_g1 was aligned with peroxiredoxin. A model peroxiredoxin (PDB ID: 3QPM) was used to perform the sequence alignment (Figure 8(a)) and 3D modeling (Figure 8(b)) of DN19197_c3_g1. Interestingly, there was no difference in the domain between DN19197_c3_g1 with the model in term of 3D modeling. Phylogenetic analysis shows that the sequence DN19197_c3_g1 is evolutionally close to the sequence AAY66580.1 from *I. scapularis* and ABY76309.1 from *I. ricinus* (Figure 8(c)).

## 4. Discussion

### 4.1. Sexual Dimorphism Exists Widely in the Same Species

The different selections between sexes lead to sexual dimorphism, and some of them serve for the sex-specific reproductive activities and requirements. Sexual dimorphism means that the female and the male show differences in natural selection, sexual selection, or nonadaptive processes. Like other animal species in nature, sexual dimorphism can influence the scorpions in the aspect of morphology, defensive behavior, venoms, and the like . The most conspicuous difference of their morphology is that the female scorpions have bigger bodies with shorter metasomal segments, whereas the bodies of male ones were smaller and their metasomal segments were longer and less rounded. This feature is directly related to the reproductive function of the females, which helps identify the sex during courtship [10, 34]. The hardness of genital operculum, the width of sternum, and the pectineous teeth in the pecten also differ between sexes. Besides, the male scorpions are faster and fed on more kinds of food, whereas the female ones are always sedentary because the newborn scorpions usually stay on the females' back and remain there for several days without eating anything [35].

Dimorphism is also considered deriving from a time-honored tradition in biometrics called allometry, which was used to study the relationship between body proportions or body shape and overall body size [36, 37]. When the trait size and body size are isometric, with every unit of the body size increases, there will be a corresponding unit of the trait size increase. Therefore, the traits scale body size. This pattern is likely in cases where fitness is directly related to trait size and, that is, a larger body size increases the ability to develop larger traits. In hypoallometry, individuals of all body sizes have more or less the same trait size. Hypoallometry may result from situations where large body size and intermediate trait size result in the highest fitness. As for hyperallometry, the large individuals express disproportionately large traits compared with smaller individuals. Hyperallometry is predicted when increasing the trait size produces greater relative fitness benefits at large body sizes. Thus, an individual in good condition devotes more resources to secondary traits than an individual in poor condition.

### 4.2. The Differentiation of Venoms between Male and Female Animals

During the process of gestation and childbirth, the females always have increases in body mass and a change of nutrient allocation, which lead to the decrease of locomotor performance and the decline of predation ability and thus increase their risk of facing natural enemies. Scorpions use their venoms for both feeding and defending, as most venomous animals do. There are also two other usage situations: for hygiene and during courtship [9]. All these differences further suggest that male and female venoms might be optimized for different tasks. Thus, the female may change the contents of their venoms to compensate for some defects. In this study, these venoms, such as potassium channel toxins, had a significant ratio of upregulation. Besides, the number of upregulated unigenes were more than that of downregulated ones in the toxin of defensins, serine protease inhibitors, sodium channel toxins, and other toxins. All these indicated that the venom of female scorpions may have stronger toxicity than that of male ones.

The variation in the component of venoms is common in other venomous animals. Study on *Loxosceles* spiders, which also can cause human injury and more than 3,000 cases of sting are reported every year, revealed that venoms from female spiders are more toxic than venoms from males in common [38]. The venoms of female *Bothrops jararaca* contained four peptide fragments, which did not exist in male snakes, and the females always had stronger lethal activity than the males [39]. Besides, the previous studies also showed that sex-biased expression existed in the venoms of other species like *Centruroides hentzi*, *Anastatus disparis*, *Brachymeria lasus*, and so on [10, 40, 41].

### 4.3. Sex is One Important Effect Factor in the Therapeutic Efficacy of Animal Medicine

As an important part of Chinese material medicine, animal medicines are widely used in clinical prescriptions and Chinese Patent Medicine. Although the varieties of animal medicines are less than those of phytomedicines, animal medicines still have great development potential. In the oldest material medical book, *Shennong's classic of material medical*, the earliest known works of traditional Chinese Medicine, a total of 65 kinds of animal medicines were recorded. Also, 128 animal medicines were included in the most complete medical book in the history of traditional Chinese medicine, *the Compendium of Materia Medica*, written by Li Shizhen, a famous medical scientist in Ming Dynasty. Overall, 50 kinds of animal medicines were included in the main part of *Chinese pharmacopoeia*, version 2015, and these animal medicines involved 76 species of medicinal animals.

The sexes of the animals, which are used as medicines, are considered not only in those ancient works but also in *Chinese pharmacopoeia*. Tubiechong, which is the dry body of female *Eupolyphaga sinensis* Walker [12], has a wide pharmacological effect, such as thrombolysis, anticoagulant, antitumor, antimutation, and hypoxia tolerance. Another animal medicine that also requires the female ones as the raw material is Oviductus Ranae, called Hamayou in Chinese. This kind of medicine is the processed products of female *Rana temporaria chensinensis David* and is widely used in antioxidant, antifatigue, antiaging, inflammation, and the like [42, 43]. On the other hand, some male animals are required as the sources of other animal medicines, such as Lurong, Lingyangjiao, and Shexiang. *Cornu cervi pantotrichum* was called Lurong in Chinese, and it was the antler of male deer, which has the potential to invigorate the spleen, strengthen bone and skeletal muscles, and promote blood flow [44]. Lingyangjiao is the horn of male *Saiga tatarica Linnaeus* and can be used to treat fever, eclampsia, and hemacelinosis [45]. Besides, Shexiang, also called artificial moschus or musk, is the dried preputial secretion of male *Moschus berezovskii Flerov*, *Moschus sifanicus Przewalski*, or *Moschus moschiferus Linnaeus*. It has the potential to be used for treating the unstable angina pectoris and neurodegenerative diseases [46].

The results of this article showed that approximately 39.53% unigenes of potassium channel toxins were upregulated in the samples of female scorpion, whereas only 16.28% were downregulated. As for metalloproteinase, there were 5 upregulated unigenes but only 1 downregulated in the female scorpions. For defensins, all the differentially expressed unigenes were upregulated. Besides, the whole ratio of differential expressed unigenes was much lower than the ratio in screened toxins, indicating that the sex-biased gene expression had more influence on the venoms than on the whole body. There is no denying that there are indeed differences in toxin composition between male and female scorpions. This study is on the level of transcriptome, and omics will be an effective method in the study of this field.

## 5. Conclusions

In this work, we first screened sequence of the MT-CO1 in the scorpions captured in Gansu Province, China, performed the phylogenetic sequence analysis, and identified the scorpions as *M. martensii*. Following *de novo* sequencing, assembly, and alignment of transcriptome, a total of 24,026 unigenes were harvested through transcriptome. We also successfully annotated 8,444 (35.15%), 7,636 (31.78%), 8,510 (35.42%), 7,840 (32.63%), 9,980 (41.54%), and 11,829 (49.23%) unigenes using the GO, KEGG, Pfam, Swissprot, eggNOG, and NR databases. Moreover, 43 metalloproteases, 40 potassium channel toxins, 24 phospholipases, 12 defensins, 10 peroxiredoxin, 9 cysteine proteinase inhibitors, 7 serine protease inhibitors, 6 sodium channel toxins, 2 NDBPs, 1 calcium channel toxin, 1 waprin-like peptides, 1 antibacterial peptide, 1 antimicrobial peptide, and 1 anticoagulant peptide were screened out. Our study discussed the sex-biased gene expression of the species *M. martensii* and their potential effect on the venoms with *de novo* transcriptomics, and the female scorpions, which consisted of more upregulated toxins, have the potential to be used as medicines with more therapeutic effective. This method can be further used as a useful way to identify the difference between female and male toxic animals.

## Figures and Tables

**Table 1:** Overview of the raw data and valid data.

Sample	Raw reads	Valid reads	Valid (%)	Q20 (%)	Q30 (%)	GC (%)
F1	51,750,108	50,424,598	97.44	96.28	89.64	42.06
F2	42,467,820	41,604,228	97.97	97.11	91.55	42.93
F3	44,188,454	43,308,140	98.01	97.10	91.49	44.98
F4	45,727,618	44,794,146	97.96	97.03	91.35	43.99
M1	45,499,340	44,613,506	98.05	96.95	91.32	41.38
M2	45,494,584	44,587,118	98.01	96.65	90.41	40.39
M3	41,850,764	40,897,900	97.72	96.40	89.92	41.23
M4	65,758,434	63,821,518	97.05	96.20	89.49	40.07

## Data Availability

The toxins screeded out can be accessed in the Supplementary Information files and the transcriptome data can be available on request through Songyu Gao (e-mail: songyu3012014@outlook.com).

## Supplementary Materials

Supplementary Materials The toxin screening data are included.
