# Supplementary material for: Sex-Biased Gene Expression of Mesobuthus martensii Collected from Gansu Province, China, Reveals Their Different Therapeutic Potentials
Source: Evid Based Complement Alternat Med. 2021 Aug 19;2021:1967158. doi: 10.1155/2021/1967158 (PMC8403048; doi:10.1155/2021/1967158)
Supplement: Supplementary Materials — The toxin screening data are included. [file 1967158.f1.docx]

**Table S1. Summary of toxins screened in the captured scorpions in transcriptome**

| **Query** | **NR** | **description** | **identity** | **swissprot** | **description** | **Identity（%）** |
| --- | --- | --- | --- | --- | --- | --- |
| **ion channel toxin** | | | | | | |
| **potassium channel toxin** | | | | | | |
| TRINITY_DN11044_c0_g1 | A0A059UEE4.1 | Full=Potassium channel toxin alpha-KTx 27.4 | 65.80 | sp\|A0A059UEE4\|KA274_MESGB | Potassium channel toxin alpha-KTx 27.4 | 65.80 |
| TRINITY_DN11874_c0_g1 | B5KF99.1 | Full=Potassium channel toxin alpha-KTx J123 | 98.40 | sp\|B5KF99\|KA11M_MESMA | Potassium channel toxin alpha-KTx J123 | 98.40 |
| TRINITY_DN13198_c0_g1 | Q9TVX3.1 | Full=Potassium channel toxin alpha-KTx 5.3; | 100.00 | sp\|Q9TVX3\|KAX53_MESMA | Potassium channel toxin alpha-KTx 5.3 | 100.00 |
| TRINITY_DN1415_c0_g1 | AIL48755.1 | potassium channel blocker pMeKTx18-3 | 79.00 | sp\|F1CIY9\|KA23H_HOTJU | U10-buthitoxin-Hj1a | 58.10 |
| TRINITY_DN14634_c0_g1 | A7KJJ7.1 | Full=Potassium channel toxin alpha-KTx 26.1 | 100.00 | sp\|A7KJJ7\|KA261_MESMA | Potassium channel blocker alpha-KTx 26.1 | 100.00 |
| TRINITY_DN14634_c0_g2 | E4VP41.1 | Full=Potassium channel toxin MeuTXKalpha4 | 82.50 | sp\|A7KJJ7\|KA261_MESMA | Potassium channel blocker alpha-KTx 26.1 | 54.00 |
| TRINITY_DN15786_c0_g1 | B8XH42.1 | Full=Potassium channel toxin alpha-KTx 16.6 | 67.70 | sp\|D3JXM1\|KA164_MESEU | Potassium channel toxin alpha-KTx 16.4 | 72.10 |
| TRINITY_DN15786_c0_g2 | Q9NBG9.1 | Full=Potassium channel toxin alpha-KTx 16.2 | 98.30 | sp\|Q9NBG9\|KA162_MESMA | Potassium channel toxin alpha-KTx 16.2 | 98.30 |
| TRINITY_DN16127_c2_g1 | K7XFK5.1 | Full=Potassium channel toxin alpha-KTx 3.16 | 88.30 | sp\|K7XFK5\|KAX3G_MESGB | Potassium channel toxin alpha-KTx 3.16 | 88.30 |
| TRINITY_DN16340_c2_g1 | B8XH44.1 | Full=Potassium channel toxin alpha-KTx 27.1 | 55.00 | sp\|A0A059UEE4\|KA274_MESGB | Potassium channel toxin alpha-KTx 27.4 | 60.30 |
| TRINITY_DN17509_c1_g5 | AIL48778.1 | potassium channel blocker pMeKTx4-1 | 96.60 | sp\|P83407\|KA191_MESMA | Potassium channel toxin alpha-KTx 19.1 | 100.00 |
| TRINITY_DN17520_c1_g1 | AMX81439.1 | potassium channel toxin meuK1 | 88.30 |  |  |  |
| TRINITY_DN17650_c1_g1 | Q95NK7.1 | Full=Potassium channel toxin alpha-KTx 14.2 | 100.00 | sp\|Q95NK7\|KA142_MESMA | Potassium channel toxin alpha-KTx 14.2 | 100.00 |
| TRINITY_DN17928_c1_g2 | XP_023241648.1 | potassium channel toxin gamma-KTx 1.1-like | 58.80 | sp\|Q86QT3\|KGX11_CENNO | Potassium channel toxin gamma-KTx 1.1 | 41.70 |
| TRINITY_DN17992_c2_g4 | Q8I0L5.1 | Full=Potassium channel toxin alpha-KTx 15.2 | 84.20 | sp\|Q8I0L5\|KA152_MESMA | Potassium channel toxin alpha-KTx 15.2 | 84.20 |
| TRINITY_DN18510_c3_g1 | Q9NII5.1 | Full=Potassium channel toxin alpha-KTx 1.6 | 70.70 | sp\|Q9NII5\|KAX16_MESMA | Potassium channel toxin alpha-KTx 1.6 | 70.70 |
| TRINITY_DN18510_c3_g2 | P13487.4 | Full=Potassium channel toxin alpha-KTx 1.1 | 74.60 | sp\|P13487\|KAX11_LEIQH | Potassium channel toxin alpha-KTx 1.1 | 74.60 |
| TRINITY_DN18510_c3_g3 | Q9NII5.1 | Full=Potassium channel toxin alpha-KTx 1.6 | 77.20 | sp\|Q9NII5\|KAX16_MESMA | Potassium channel toxin alpha-KTx 1.6 | 77.20 |
| TRINITY_DN18510_c3_g4 | Q9NII5.1 | Full=Potassium channel toxin alpha-KTx 1.6 | 100.00 | sp\|Q9NII5\|KAX16_MESMA | Potassium channel toxin alpha-KTx 1.6 | 100.00 |
| TRINITY_DN18769_c2_g5 | Q9NJC6.1 | Full=Potassium channel toxin BmTXK-beta | 97.00 | sp\|Q9NJC6\|KBX2_MESMA | Potassium channel toxin BmTXK-beta | 97.00 |
| TRINITY_DN18769_c2_g6 | Q9NJC6.1 | Full=Potassium channel toxin BmTXK-beta | 100.00 | sp\|Q9NJC6\|KBX2_MESMA | Potassium channel toxin BmTXK-beta | 100.00 |
| TRINITY_DN19000_c1_g4 | Q9NII6.1 | Full=Potassium channel toxin alpha-KTx 1.5 | 100.00 | sp\|Q9NII6\|KAX15_MESMA | Potassium channel toxin alpha-KTx 1.5 | 100.00 |
| TRINITY_DN19286_c0_g2 | AIL48798.1 | potassium channel blocker pMeKTx30-1 | 84.10 | sp\|B8XH22\|SCX8_BUTOS | Putative calcium channel toxin Tx758 | 54.10 |
| TRINITY_DN19626_c3_g2 | Q9N661.1 | Full=Potassium channel toxin BmTXK-beta-2 | 100.00 | sp\|Q9N661\|KBX1_MESMA | Potassium channel toxin BmTXK-beta-2 | 100.00 |
| TRINITY_DN20075_c1_g4 | AIL48765.1 | potassium channel blocker pMeKTx7-1 | 87.50 | sp\|Q95NJ8\|KA171_MESMA | Potassium channel toxin alpha-KTx 17.1 | 72.50 |
| TRINITY_DN20164_c0_g1 | Q95NJ8.1 | Full=Potassium channel toxin alpha-KTx 17.1 | 98.20 | sp\|Q95NJ8\|KA171_MESMA | Potassium channel toxin alpha-KTx 17.1 | 98.20 |
| TRINITY_DN20164_c1_g2 | Q95NJ8.1 | Full=Potassium channel toxin alpha-KTx 17.1 | 76.40 | sp\|Q95NJ8\|KA171_MESMA | Potassium channel toxin alpha-KTx 17.1 | 76.40 |
| TRINITY_DN20276_c0_g1 | Q9U8D1.1 | Full=Potassium channel toxin alpha-KTx 9.2 | 100.00 | sp\|Q9U8D1\|KAX92_MESMA | Potassium channel toxin alpha-KTx 9.2 | 100.00 |
| TRINITY_DN20512_c1_g1 | AIL48780.1 | potassium channel blocker pMeKTx27-1 | 77.40 | sp\|B8XH30\|KA23K_BUTOS | Potassium channel toxin alpha-KTx Tx308 | 72.10 |
| TRINITY_DN20512_c1_g2 | AAM94410.1 | potassium channel blocker BmK38 precursor | 100.00 | sp\|Q8MUB1\|KA221_MESMA | Potassium channel toxin alpha-KTx 22.1 | 96.80 |
| TRINITY_DN21180_c0_g1 | Q9NII7.1 | Full=Potassium channel toxin alpha-KTx 3.6 | 100.00 | sp\|Q9NII7\|KAX36_MESMA | Potassium channel toxin alpha-KTx 3.6 | 100.00 |
| TRINITY_DN36_c0_g1 | B8XH38.1 | Full=Potassium channel toxin-like Tx677 | 72.10 | sp\|B8XH38\|KAX_BUTOS | Potassium channel toxin-like Tx677 | 72.10 |
| TRINITY_DN6951_c0_g1 | P59938.1 | Full=Potassium channel toxin gamma-KTx 2.2 | 100.00 | sp\|P59938\|KGX22_MESMA | Potassium channel toxin gamma-KTx 2.2 | 100.00 |
| TRINITY_DN7419_c0_g1 | B8XH44.1 | Full=Potassium channel toxin alpha-KTx 27.1 | 86.40 | sp\|B8XH44\|KA271_BUTOS | Potassium channel toxin alpha-KTx 27.1 | 86.40 |
| TRINITY_DN20580_c2_g1 | AAF24057.1 | neurotoxin P01 | 100.00 | sp\|Q9U8D2\|KAX82_MESMA | Potassium channel toxin alpha-KTx 8.2 | 96.50 |
| TRINITY_DN18396_c3_g2 | ACJ23158.1 | putative toxin Tx277 | 76.20 | sp\|P0DL62\|KA311_BUTOC | Potassium channel toxin alpha-KTx 31.1 | 65.80 |
| **Na channel toxin** | | | | | | |
| TRINITY_DN11226_c0_g1 | M1J7U4.1 | Full=Putative sodium channel alpha-toxin Acra5 | 84.80 | sp\|M1J7U4\|SCX5_ANDCR | Putative sodium channel alpha-toxin Acra5 | 84.80 |
| TRINITY_DN17928_c1_g1 | ABR21048.1 | venom sodium channel toxin-4 | 91.90 |  |  |  |
| TRINITY_DN17949_c2_g1 | AMX81461.1 | sodium channel toxin meuNa10 | 78.70 | sp\|P24336\|SIX2_HOTJU | Beta-insect depressant toxin BjIT2 | 65.60 |
| TRINITY_DN17949_c2_g2 | AMX81453.1 | sodium channel toxin meuNa6 | 71.60 | sp\|Q8I0K7\|SIXI_MESMA | Depressant scorpion toxin BmKIM | 52.20 |
| TRINITY_DN19326_c3_g3 | ADT82854.1 | sodium channel neurotoxin alpha type NaTxalpha-12, partial | 82.90 | sp\|P60255\|SCXA_BUTOM | Toxin Boma6a | 75.60 |
| **Ca channel toxin** | | | | | | |
| TRINITY_DN17597_c2_g2 | AAZ16270.1 | putative Ca channel inhibitor | 100.00 | sp\|Q8I6X9\|SCXC1_MESMA | Toxin BmCa-1 | 68.30 |
| **host defense peptides** | | | | | | |
| **NDBPs** | | | | | | |
| TRINITY_DN19670_c0_g3 | Q9GQW4.1 | Full=Peptide BmKn1 | 100.00 | sp\|Q9GQW4\|NDB4U_MESMA | Peptide BmKn1 | 100.00 |
| TRINITY_DN20805_c0_g30 | Q8N0N8.1 | Full=Peptide BmKa2 | 100.00 | sp\|Q8N0N8\|NDB2S_MESMA | Peptide BmKa2 | 100.00 |
| **defensins** | | | | | | |
| TRINITY_DN11679_c0_g1 | AIX87626.1 | defensin-1 | 72.10 | sp\|P41965\|DEF4_LEIQH | 4 kDa defensin | 94.70 |
| TRINITY_DN14118_c0_g9 | AIX87626.1 | defensin-1 | 64.80 |  |  |  |
| TRINITY_DN14345_c0_g2 | AHZ63116.1 | defensin-like protein, partial | 98.40 |  |  |  |
| TRINITY_DN14804_c0_g1 | XP_023234642.1 | putative defensin-like protein 66 | 45.60 |  |  |  |
| TRINITY_DN14804_c0_g2 | XP_023234642.1 | putative defensin-like protein 66 | 75.80 |  |  |  |
| TRINITY_DN14979_c0_g2 | AAK57517.1 | defensin-like protein TXKS2 | 96.70 | sp\|Q95P88\|DEF_MESMA | Defensin-like peptide TXKs2 | 96.70 |
| TRINITY_DN20242_c5_g2 | AIX87624.1 | defensin-4 | 81.80 |  |  |  |
| TRINITY_DN20775_c0_g2 | XP_023234642.1 | putative defensin-like protein 66 | 61.10 |  |  |  |
| TRINITY_DN9071_c0_g1 | AIX87626.1 | defensin-1 | 82.00 | sp\|Q86QI5\|DEF1_DERVA | Defensin | 70.00 |
| TRINITY_DN9071_c0_g2 | AIX87626.1 | defensin-1 | 85.50 | sp\|Q86LE4\|DEFI_RHIMP | Defensin | 55.40 |
|  |  |  |  | sp\|Q40901\|DEF_PETIN | Defensin-like protein | 69.20 |
|  |  |  |  | sp\|Q40901\|DEF_PETIN | Defensin-like protein | 74.40 |
| **waprin-like peptides** | | | | | | |
| TRINITY_DN20408_c0_g1 | XP_023240400.1 | waprin-Enh1-like | 84.30 |  |  |  |
| **putative enzymes and protease inhibitors** | | | | | | |
| **metalloprotease** | | | | | | |
| TRINITY_DN10730_c0_g1 | XP_023212189.1 | astacin-like metalloprotease toxin 5 | 75.30 |  |  |  |
| TRINITY_DN10730_c0_g2 | XP_023212189.1 | astacin-like metalloprotease toxin 5 | 71.30 | sp\|P07584\|ASTA_ASTAS | Astacin | 45.50 |
| TRINITY_DN14729_c0_g1 | ADY39557.1 | M12B metalloprotease, partial | 65.40 | sp\|V9ZAY0\|VMPA1_TITTR | Venom metalloproteinase antarease-like TtrivMP_A | 35.60 |
| TRINITY_DN15207_c0_g1 | ABR20110.1 | venom metalloprotease-1 | 33.60 | sp\|V9ZAY0\|VMPA1_TITTR | Venom metalloproteinase antarease-like TtrivMP_A | 41.90 |
| TRINITY_DN18932_c0_g1 | ABR20110.1 | venom metalloprotease-1 | 27.40 |  |  |  |
| TRINITY_DN19942_c0_g1 | ABR20110.1 | venom metalloprotease-1 | 91.40 | sp\|V9ZAY0\|VMPA1_TITTR | Venom metalloproteinase antarease-like TtrivMP_A | 40.10 |
| TRINITY_DN20073_c0_g1 | ADY39479.1 | putative M12B metalloprotease, partial | 61.90 | sp\|V9ZAY0\|VMPA1_TITTR | Venom metalloproteinase antarease-like TtrivMP_A | 49.20 |
| TRINITY_DN10108_c0_g1 | XP_023238792.1 | MAM and LDL-receptor class A domain-containing protein 1-like | 73.50 | sp\|Q9N2V2\|NAS30_CAEEL | Zinc metalloproteinase nas-30 | 43.50 |
| TRINITY_DN12875_c0_g2 | XP_023217139.1 | inducible metalloproteinase inhibitor protein-like isoform X1 | 79.10 |  |  |  |
| TRINITY_DN13458_c0_g1 | XP_023212236.1 | venom metalloproteinase antarease-like TtrivMP_A | 43.80 | sp\|V9ZAY0\|VMPA1_TITTR | Venom metalloproteinase antarease-like TtrivMP_A | 39.80 |
| TRINITY_DN13489_c0_g4 | XP_023231982.1 | matrix metalloproteinase-2-like | 78.90 | sp\|Q8MPP3\|D2MP_DROME | Matrix metalloproteinase-2 | 49.50 |
| TRINITY_DN13659_c0_g1 | CDJ26728.1 | MAM-containing Astacin-like metallopeptidase protein | 62.80 | sp\|A0FKN6\|VMPA_LOXIN | Astacin-like metalloprotease toxin 1 | 41.60 |
| TRINITY_DN15486_c0_g1 | XP_023234508.1 | venom metalloproteinase antarease-like TtrivMP_A | 42.70 | sp\|V9ZAY0\|VMPA1_TITTR | Venom metalloproteinase antarease-like TtrivMP_A | 38.40 |
| TRINITY_DN16917_c1_g2 | XP_023240395.1 | metalloproteinase inhibitor 3-like isoform X3 | 67.10 |  |  |  |
| TRINITY_DN17274_c2_g3 | XP_023224475.1 | tissue inhibitor of metalloproteinase-like isoform X1 | 66.50 |  |  |  |
| TRINITY_DN16094_c0_g1 | AMO02513.1 | A disintegrin and metalloproteinase 1 | 81.20 | sp\|O43184\|ADA12_HUMAN | Disintegrin and metalloproteinase domain-containing protein 12 | 37.10 |
| TRINITY_DN16300_c4_g3 | XP_023225572.1 | LOW QUALITY PROTEIN: putative zinc metalloproteinase YIL108W | 66.70 | sp\|Q9US12\|YK66_SCHPO | Putative zinc metalloproteinase C607.06c | 28.40 |
| TRINITY_DN16345_c6_g12 | XP_023243347.1 | disintegrin and metalloproteinase domain-containing protein 10-like | 87.30 | sp\|Q10741\|ADA10_BOVIN | Disintegrin and metalloproteinase domain-containing protein 10 | 46.10 |
| TRINITY_DN17694_c2_g2 | XP_023234399.1 | A disintegrin and metalloproteinase with thrombospondin motifs 9-like | 80.80 | sp\|Q9P2N4\|ATS9_HUMAN | A disintegrin and metalloproteinase with thrombospondin motifs 9 | 34.60 |
| TRINITY_DN17694_c2_g5 | XP_023234399.1 | A disintegrin and metalloproteinase with thrombospondin motifs 9-like | 76.10 |  |  |  |
| TRINITY_DN17894_c0_g1 | AMO02542.1 | astacin-like protein metallopeptidase 20 | 52.30 | sp\|A0FKN6\|VMPA_LOXIN | Astacin-like metalloprotease toxin 1 | 38.60 |
| TRINITY_DN17894_c0_g5 | AMO02542.1 | astacin-like protein metallopeptidase 20 | 59.10 | sp\|A0FKN6\|VMPA_LOXIN | Astacin-like metalloprotease toxin 1 | 44.70 |
| TRINITY_DN20507_c2_g5 | XP_023234770.1 | venom metalloproteinase 3-like isoform X1 | 91.40 | sp\|Q9R001\|ATS5_MOUSE | A disintegrin and metalloproteinase with thrombospondin motifs 5 | 29.00 |
| TRINITY_DN9612_c0_g1 | XP_023211289.1 | disintegrin and metalloproteinase domain-containing protein 10-like | 42.70 |  |  |  |
| TRINITY_DN10849_c0_g1 | XP_023216275.1 | uncharacterized protein LOC111618883 | 50.00 | sp\|P58397\|ATS12_HUMAN | A disintegrin and metalloproteinase with thrombospondin motifs 12 | 27.20 |
| TRINITY_DN15033_c0_g1 | XP_023226830.1 | uncharacterized protein LOC111627483 | 100.00 | sp\|Q10741\|ADA10_BOVIN | Disintegrin and metalloproteinase domain-containing protein 10 | 59.30 |
| TRINITY_DN15949_c3_g3 | AHA36326.1 | acid trehalase | 43.30 | sp\|V9ZAY0\|VMPA1_TITTR | Venom metalloproteinase antarease-like TtrivMP_A | 28.70 |
| TRINITY_DN15949_c3_g4 | AHA36326.1 | acid trehalase | 68.70 | sp\|V9ZAY0\|VMPA1_TITTR | Venom metalloproteinase antarease-like TtrivMP_A | 48.40 |
| TRINITY_DN16944_c1_g9 | XP_023215974.1 | uncharacterized protein LOC111618648 | 79.10 | sp\|Q9R1V4\|ADA11_MOUSE | Disintegrin and metalloproteinase domain-containing protein 11 | 35.10 |
| TRINITY_DN18323_c3_g1 | AHA36326.1 | acid trehalase | 37.30 | sp\|V9ZAX6\|VMPA2_TITPA | Venom metalloproteinase antarease-like TpachMP_B (Fragment) | 28.00 |
| TRINITY_DN18323_c3_g2 | AHA36326.1 | acid trehalase | 39.20 | sp\|V9ZAY0\|VMPA1_TITTR | Venom metalloproteinase antarease-like TtrivMP_A | 31.10 |
| TRINITY_DN19835_c0_g3 | XP_023234263.1 | sterile alpha and TIR motif-containing protein 1-like | 63.90 | sp\|Q8MPP3\|D2MP_DROME | Matrix metalloproteinase-2 | 39.20 |
| TRINITY_DN18932_c0_g2 | AHA36326.1 | acid trehalase | 100.00 | sp\|V9ZAY0\|VMPA1_TITTR | Venom metalloproteinase antarease-like TtrivMP_A | 45.50 |
| TRINITY_DN19942_c0_g2 | AHA36326.1 | acid trehalase | 59.70 | sp\|P86392\|VMPA1_TITSE | Venom metalloproteinase antarease TserMP_A (Fragment) | 40.30 |
| TRINITY_DN19942_c0_g3 | AHA36326.1 | acid trehalase | 64.80 | sp\|V9ZAY0\|VMPA1_TITTR | Venom metalloproteinase antarease-like TtrivMP_A | 38.00 |
| TRINITY_DN20553_c2_g2 | AHA36326.1 | acid trehalase | 77.70 | sp\|V9ZAY0\|VMPA1_TITTR | Venom metalloproteinase antarease-like TtrivMP_A | 35.30 |
| TRINITY_DN20553_c2_g3 | AHA36326.1 | acid trehalase | 85.90 | sp\|V9ZAY0\|VMPA1_TITTR | Venom metalloproteinase antarease-like TtrivMP_A | 47.20 |
| TRINITY_DN8241_c0_g1 | AHA36326.1 | acid trehalase | 41.80 | sp\|V9ZAY0\|VMPA1_TITTR | Venom metalloproteinase antarease-like TtrivMP_A | 33.60 |
| **phospholipases** | | | | | | |
| TRINITY_DN14893_c0_g1 | XP_023238662.1 | phospholipase D3-like isoform X3 | 87.10 | sp\|Q8BG07\|PLD4_MOUSE | Phospholipase D4 | 42.40 |
| TRINITY_DN15447_c1_g1 | XP_023217158.1 | phospholipase D3-like isoform X2 | 82.70 | sp\|Q6PB03\|PLD3_XENLA | Phospholipase D3 | 50.00 |
| TRINITY_DN16213_c0_g1 | XP_023236868.1 | acidic phospholipase A2 PA4-like | 47.10 | sp\|Q7M4I5\|PA2_APIDO | Phospholipase A2 | 46.90 |
| TRINITY_DN16678_c0_g1 | XP_023234369.1 | phospholipase A2-like isoform X2 | 51.50 | sp\|P00630\|PA2_APIME | Phospholipase A2 | 43.70 |
| TRINITY_DN17691_c5_g1 | XP_023217052.1 | group XV phospholipase A2-like isoform X1 | 83.70 | sp\|Q6XPZ3\|PAG15_CANLF | Group XV phospholipase A2 | 50.00 |
| TRINITY_DN17933_c0_g2 | XP_023235769.1 | phospholipase A-2-activating protein-like isoform X2 | 92.10 | sp\|P27612\|PLAP_MOUSE | Phospholipase A-2-activating protein | 51.20 |
| TRINITY_DN19085_c1_g6 | XP_002406591.1 | phospholipase C gamma, putative | 69.30 | sp\|P19174\|PLCG1_HUMAN | 1-phosphatidylinositol 4,5-bisphosphate phosphodiesterase gamma-1 | 52.20 |
| TRINITY_DN19180_c0_g1 | XP_023234380.1 | phospholipase A2-like isoform X1 | 66.20 | sp\|P00630\|PA2_APIME | Phospholipase A2 | 43.80 |
| TRINITY_DN19180_c0_g4 | XP_023234380.1 | phospholipase A2-like isoform X1 | 63.30 | sp\|Q7M4I5\|PA2_APIDO | Phospholipase A2 | 43.70 |
| TRINITY_DN18013_c3_g1 | XP_023222756.1 | phospholipase D SdSicTox-betaIIB1bxii-like | 66.60 | sp\|Q1W694\|B1Q_LOXIN | Phospholipase D LiSicTox-betaID1 | 47.60 |
| TRINITY_DN18603_c2_g2 | XP_023221920.1 | phospholipase ABHD3-like | 89.70 | sp\|Q91ZH7\|ABHD3_MOUSE | Phospholipase ABHD3 | 37.90 |
| TRINITY_DN15844_c1_g17 | XP_023229534.1 | group XIIA secretory phospholipase A2-like | 74.00 |  |  |  |
| TRINITY_DN16436_c1_g1 | XP_023225358.1 | lysophospholipase D GDPD1-like | 87.60 |  |  |  |
| TRINITY_DN18316_c2_g3 | XP_023237656.1 | phospholipase D2-like isoform X1 | 69.70 |  |  |  |
| TRINITY_DN19184_c1_g8 | ADY39496.1 | putative phospholipase C, partial | 95.70 |  |  |  |
| TRINITY_DN19225_c2_g2 | XP_023234247.1 | putative phospholipase B-like 2 | 82.20 | sp\|Q3TCN2\|PLBL2_MOUSE | Putative phospholipase B-like 2 | 51.80 |
| TRINITY_DN19272_c2_g1 | XP_023219823.1 | patatin-like phospholipase domain-containing protein 7 | 61.40 | sp\|A2AJ88\|PLPL7_MOUSE | Patatin-like phospholipase domain-containing protein 7 | 58.20 |
| TRINITY_DN19368_c1_g1 | XP_023234369.1 | phospholipase A2-like isoform X2 | 48.10 | sp\|P00630\|PA2_APIME | Phospholipase A2 | 43.70 |
| TRINITY_DN20578_c0_g3 | XP_023210300.1 | N-acyl-phosphatidylethanolamine-hydrolyzing phospholipase D-like isoform X1 | 89.60 | sp\|Q6IQ20\|NAPEP_HUMAN | N-acyl-phosphatidylethanolamine-hydrolyzing phospholipase D | 63.30 |
| TRINITY_DN20644_c0_g6 | XP_023221513.1 | patatin-like phospholipase domain-containing protein 2 | 84.10 | sp\|Q8BJ56\|PLPL2_MOUSE | Patatin-like phospholipase domain-containing protein 2 | 43.30 |
| TRINITY_DN21220_c0_g1 | XP_023231606.1 | phospholipase A2, membrane associated-like | 76.10 | sp\|D2X8K2\|PA2_CONGI | Phospholipase A2 A2-actitoxin-Cgg2a | 39.80 |
| TRINITY_DN13477_c0_g1 | XP_023225236.1 | 85/88 kDa calcium-independent phospholipase A2-like isoform X1 | 86.70 | sp\|O60733\|PLPL9_HUMAN | 85/88 kDa calcium-independent phospholipase A2 | 44.60 |
| TRINITY_DN19072_c1_g3 | XP_023211072.1 | calcium-independent phospholipase A2-gamma-like isoform X1 | 77.60 | sp\|Q9NP80\|PLPL8_HUMAN | Calcium-independent phospholipase A2-gamma | 47.10 |
| TRINITY_DN17138_c2_g1 | XP_023242353.1 | uncharacterized protein LOC111640561, partial | 78.30 | sp\|Q7M4I6\|PA2_BOMPE | Phospholipase A2 | 48.90 |
| **serine proteases and serine protease inhibitors** | | | | | | |
| TRINITY_DN12531_c0_g1 | P0DJ50.2 | Full=Kunitz-type serine protease inhibitor BmKTT-2 | 100.00 | sp\|P0DJ50\|VKT31_MESMA | Kunitz-type serine protease inhibitor BmKTT-2 | 100.00 |
| TRINITY_DN18666_c0_g2 | ABR20123.1 | venom serine protease inhibitor, partial | 95.50 |  |  |  |
| TRINITY_DN2119_c0_g1 | XP_023222120.1 | kunitz-type serine protease inhibitor-like | 75.70 | sp\|D4A2Z2\|EPPI_RAT | Eppin | 48.30 |
| TRINITY_DN2773_c0_g1 | P0DJ47.1 | Full=Kunitz-type serine protease inhibitor BmKTT-3 | 100.00 | sp\|P0DJ47\|VKT12_MESMA | Kunitz-type serine protease inhibitor BmKTT-3 | 100.00 |
| TRINITY_DN14906_c0_g1 | AIX87608.1 | Kunitz-type carboxypeptidase inhibitor Kci-1 | 68.30 | sp\|P0DJ50\|VKT31_MESMA | Kunitz-type serine protease inhibitor BmKTT-2 | 55.80 |
| TRINITY_DN2102_c0_g1 | AIX87608.1 | Kunitz-type carboxypeptidase inhibitor Kci-1 | 69.70 | sp\|P0DJ50\|VKT31_MESMA | Kunitz-type serine protease inhibitor BmKTT-2 | 65.20 |
| TRINITY_DN16047_c1_g1 | ALX72370.1 | venom protein VP5 | 91.20 | sp\|P0DJ49\|VKT24_MESMA | Kunitz-type serine protease inhibitor BmKTT-1 | 100.00 |
| **Cysteine proteinase inhibitor** | | | | | | |
| TRINITY_DN18863_c1_g1 | XP_023244832.1 | cysteine proteinase inhibitor 8-like isoform X2 | 54.30 |  |  |  |
| TRINITY_DN20786_c0_g1 | XP_015929473.1 | digestive cysteine proteinase 2-like | 59.40 | sp\|Q54TR1\|CFAD_DICDI | Counting factor associated protein D | 40.90 |
| TRINITY_DN3026_c0_g1 | ACR56863.1 | cathepsin L-like cysteine proteinase | 55.70 | sp\|O65039\|CYSEP_RICCO | Vignain | 86.30 |
| TRINITY_DN8059_c0_g1 | KRY02207.1 | Cysteine proteinase inhibitor 6, partial | 59.40 | sp\|Q06445\|CYTI_VIGUN | Cysteine proteinase inhibitor | 67.70 |
| TRINITY_DN12485_c0_g1 | CDS26432.1 | Kunitz protease inhibitor | 62.50 | sp\|P0DJ50\|VKT31_MESMA | Kunitz-type serine protease inhibitor BmKTT-2 | 56.10 |
| TRINITY_DN9902_c0_g1 | XP_015795816.1 | cathepsin B-like | 65.30 | sp\|P43510\|CPR6_CAEEL | Cathepsin B-like cysteine proteinase 6 | 60.50 |
| TRINITY_DN20581_c0_g1 | XP_023238299.1 | cathepsin L-like | 77.90 | sp\|Q9VN93\|CPR1_DROME | Putative cysteine proteinase CG12163 | 46.70 |
| TRINITY_DN14616_c0_g1 | XP_015667373.1 | PREDICTED: pro-cathepsin H | 55.00 | sp\|Q10717\|CYSP2_MAIZE | Cysteine proteinase 2 | 75.60 |
| TRINITY_DN12978_c0_g1 | XP_003724610.1 | PREDICTED: cathepsin L1 | 45.70 | sp\|P43297\|RD21A_ARATH | Cysteine proteinase RD21A | 66.40 |
| **peroxiredoxin** | | | | | | |
| TRINITY_DN11490_c0_g1 | XP_023223106.1 | peroxiredoxin 1-like isoform X1 | 93.40 | sp\|Q9V3P0\|PRDX1_DROME | Peroxiredoxin 1 | 75.90 |
| TRINITY_DN14810_c0_g1 | XP_023233090.1 | peroxiredoxin-2-like | 84.80 | sp\|P20108\|PRDX3_MOUSE | Thioredoxin-dependent peroxide reductase, mitochondrial | 70.40 |
| TRINITY_DN16690_c1_g1 | XP_023234227.1 | peroxiredoxin-6-like | 88.60 | sp\|O77834\|PRDX6_BOVIN | Peroxiredoxin-6 | 66.10 |
| TRINITY_DN19197_c3_g1 | XP_023219033.1 | peroxiredoxin-4-like | 88.50 | sp\|Q13162\|PRDX4_HUMAN | Peroxiredoxin-4 | 72.40 |
| TRINITY_DN19468_c0_g1 | XP_023217763.1 | peroxiredoxin-5, mitochondrial-like | 80.30 | sp\|P99029\|PRDX5_MOUSE | Peroxiredoxin-5, mitochondrial | 58.00 |
| TRINITY_DN21330_c0_g1 | NP_001027191.1 | peroxiredoxin 5, isoform B | 100.00 | sp\|Q9GLW7\|PRDX5_CHLAE | Peroxiredoxin-5, mitochondrial | 61.50 |
| TRINITY_DN4302_c0_g1 | XP_014468602.1 | PREDICTED: peroxiredoxin-5, mitochondrial isoform X1 | 61.20 | sp\|Q9XEX2\|PRX2B_ARATH | Peroxiredoxin-2B | 82.10 |
| TRINITY_DN69_c0_g1 | XP_021084022.1 | peroxiredoxin-1 | 93.40 | sp\|P35700\|PRDX1_MOUSE | Peroxiredoxin-1 | 100.00 |
| TRINITY_DN9279_c0_g1 | XP_017558880.1 | PREDICTED: peroxiredoxin-1 | 98.70 | sp\|Q91191\|TDX_ONCMY | Peroxiredoxin | 88.20 |
| TRINITY_DN6133_c0_g1 | NP_477510.1 | thioredoxin peroxidase 1, isoform A | 100.00 | sp\|Q9V3P0\|PRDX1_DROME | Peroxiredoxin 1 | 100.00 |
| **other venoms** | | | | | | |
| TRINITY_DN10176_c0_g1 | XP_023215422.1 | toxin-like protein | 69.90 |  |  |  |
| TRINITY_DN1029_c0_g1 | XP_023237471.1 | toxin CSTX-20-like | 36.10 |  |  |  |
| TRINITY_DN10436_c0_g1 | AAK61818.1 | putative toxin-like peptide KTXLP2 | 95.20 | sp\|Q95P90\|LV1B_MESMA | HMG-CoA reductase inhibitor bumarsin | 95.20 |
| TRINITY_DN10736_c0_g2 | XP_023234522.1 | uncharacterized protein LOC111634077 isoform X1 | 41.80 | sp\|P0CJ08\|VP29_LYCMC | Venom protein 29 | 32.50 |
| TRINITY_DN10738_c0_g1 | ABR21057.1 | venom protein-6 | 81.40 |  |  |  |
| TRINITY_DN10813_c0_g1 | XP_023234740.1 | venom protein 302-like | 64.80 |  |  |  |
| TRINITY_DN11226_c0_g2 | E7BLC7.1 | Full=Toxin Acra3 | 69.00 | sp\|E7BLC7\|TX30_ANDCR | Toxin Acra3 | 69.00 |
| TRINITY_DN11647_c0_g1 | XP_023232240.1 | venom protein 164-like | 64.20 |  |  |  |
| TRINITY_DN12059_c0_g1 | AGV98853.1 | androcin | 83.20 | sp\|P0CJ14\|VP302_LYCMC | Venom protein 302 | 40.40 |
| TRINITY_DN12398_c0_g1 | ADY39581.1 | U1-buthitoxin-Hj1b | 67.10 |  |  |  |
| TRINITY_DN12528_c0_g1 | AOF40178.1 | venom peptide HtC6Tx3 | 58.20 |  |  |  |
| TRINITY_DN12766_c0_g1 | XP_023241814.1 | venom protein 302-like | 52.40 |  |  |  |
| TRINITY_DN12842_c0_g1 | XP_023212190.1 | venom peptide MmKTx1-like | 79.40 |  |  |  |
|  |  |  |  | sp\|O65740\|DEF2_CAPAN | Defensin J1-2 | 50.00 |
| TRINITY_DN1295_c0_g1 | XP_023217448.1 | venom protein 302-like | 77.00 |  |  |  |
| TRINITY_DN13213_c0_g1 | Q26292.1 | Full=Beta-insect depressant toxin LqhIT2 | 83.50 | sp\|Q26292\|SIX2_LEIQH | Beta-insect depressant toxin LqhIT2 | 83.50 |
| TRINITY_DN13481_c0_g1 | ABR21080.1 | venom lipolysis activating peptide alpha subunit | 48.50 |  |  |  |
| TRINITY_DN14068_c0_g2 | AIX87724.1 | orphan peptide AbOp-5 | 93.40 | sp\|P0CJ12\|VP59_LYCMC | Venom protein 59.1 | 46.50 |
| TRINITY_DN14075_c0_g1 | XP_023211867.1 | plancitoxin-1-like isoform X1 | 69.20 | sp\|O62855\|DNS2A_PIG | Deoxyribonuclease-2-alpha | 33.80 |
| TRINITY_DN14307_c0_g1 | XP_023238420.1 | U24-ctenitoxin-Pn1a-like | 76.50 |  |  |  |
| TRINITY_DN14625_c0_g1 | XP_023218281.1 | venom phosphodiesterase 2-like | 79.00 | sp\|P15396\|ENPP3_BOVIN | Ectonucleotide pyrophosphatase/phosphodiesterase family member 3 | 38.00 |
| TRINITY_DN14647_c0_g1 | AIX87811.1 | venom protein AbVp-16, partial | 77.40 |  |  |  |
| TRINITY_DN14706_c0_g1 | AMX81469.1 | venom peptide meuPep31 | 75.00 |  |  |  |
| TRINITY_DN1534_c0_g1 | ABR20122.1 | venom protein | 95.70 |  |  |  |
| TRINITY_DN15356_c0_g2 | XP_023209523.1 | toxin Acra I-2-like | 44.90 |  |  |  |
| TRINITY_DN1542_c0_g1 | XP_023217418.1 | venom protein 302-like | 57.30 | sp\|P0CJ14\|VP302_LYCMC | Venom protein 302 | 50.00 |
| TRINITY_DN15630_c4_g3 | Q6WJF5.3 | Full=Lipolysis-activating peptide 1-alpha chain | 91.40 | sp\|Q6WJF5\|LV1A_MESMA | Lipolysis-activating peptide 1-alpha chain | 91.40 |
| TRINITY_DN16062_c2_g1 | XP_023212351.1 | cysteine-rich venom protein LEI1-like | 88.80 | sp\|Q2XXQ6\|CRVP1_DISTY | Cysteine-rich venom protein DIS1 | 37.30 |
| TRINITY_DN16430_c0_g3 | XP_023234524.1 | uncharacterized protein LOC111634077 isoform X3 | 39.20 | sp\|P0CJ08\|VP29_LYCMC | Venom protein 29 | 22.30 |
| TRINITY_DN16450_c1_g13 | ABR21046.1 | venom toxin-like peptide-6 | 97.20 |  |  |  |
| TRINITY_DN16457_c0_g5 | ABR21071.1 | venom protein-5 | 96.50 |  |  |  |
| TRINITY_DN16511_c3_g3 | XP_023234363.1 | venom protein 302-like | 59.00 |  |  |  |
| TRINITY_DN16511_c3_g4 | XP_023234363.1 | venom protein 302-like | 53.50 |  |  |  |
| TRINITY_DN16745_c1_g1 | XP_023233208.1 | 5'-nucleotidase-like | 79.50 | sp\|F8S0Z7\|V5NTD_CROAD | Snake venom 5'-nucleotidase | 43.70 |
| TRINITY_DN16863_c0_g1 | XP_023229085.1 | venom protein 7.1-like | 36.20 |  |  |  |
| TRINITY_DN16928_c2_g2 | AEI61921.1 | alpha-toxin, partial | 36.50 | sp\|P60212\|SCX2_TITOB | Alpha-toxin To2 | 37.30 |
| TRINITY_DN16965_c4_g1 | XP_023234355.1 | venom protein 302-like | 63.10 | sp\|Q7T3Q2\|CRIM1_DANRE | Cysteine-rich motor neuron 1 protein | 38.50 |
| TRINITY_DN16981_c4_g2 | XP_023241578.1 | venom allergen 5-like | 70.70 | sp\|W4VS53\|CRVP_TRILK | CRISP/Allergen/PR-1 | 39.80 |
| TRINITY_DN17169_c2_g2 | Q9UAC9.1 | Full=Beta-toxin BmKAS | 98.60 | sp\|Q9UAC9\|SCAS_MESMA | Beta-toxin BmKAS | 98.60 |
| TRINITY_DN17284_c1_g3 | API81349.1 | venom toxin | 55.90 |  |  |  |
| TRINITY_DN17284_c1_g7 | API81349.1 | venom toxin | 53.50 | sp\|P0CJ14\|VP302_LYCMC | Venom protein 302 | 53.70 |
| TRINITY_DN17387_c0_g1 | Q9NBW2.1 | Full=Toxin BmKBT | 100.00 | sp\|Q9NBW2\|SCBT_MESMA | Toxin BmKBT | 100.00 |
| TRINITY_DN17581_c1_g3 | Q9UAC8.1 | Full=Beta-toxin BmKAs1 | 100.00 | sp\|Q9UAC8\|SCAT_MESMA | Beta-toxin BmKAs1 | 100.00 |
| TRINITY_DN17629_c3_g1 | P0CJ14.1 | Full=Venom protein 302 | 56.00 | sp\|P0CJ14\|VP302_LYCMC | Venom protein 302 | 56.00 |
| TRINITY_DN17647_c0_g1 | Q9Y1U3.2 | Full=Toxin BmKITc | 95.50 | sp\|Q9Y1U3\|SIXC_MESMA | Toxin BmKITc | 95.50 |
| TRINITY_DN17686_c2_g2 | XP_023223593.1 | toxin CSTX-20-like | 57.00 |  |  |  |
| TRINITY_DN17933_c1_g1 | AMX81465.1 | venom toxin meuEnz25 | 97.80 | sp\|O14548\|COX7R_HUMAN | Cytochrome c oxidase subunit 7A-related protein, mitochondrial | 41.90 |
|  |  |  |  | sp\|Q9Y0X5\|NDBX_MESMA | Peptide BmKa1 | 98.20 |
| TRINITY_DN18248_c1_g1 | AMX81479.1 | venom peptide meuPep34 | 83.50 |  |  |  |
| TRINITY_DN18272_c0_g1 | ALX72371.1 | venom protein VP6 | 93.40 |  |  |  |
| TRINITY_DN18290_c2_g1 | XP_023241070.1 | venom protein 54.1-like | 49.60 | sp\|P0CJ07\|VP54_LYCMC | Venom protein 54.1 | 49.10 |
| TRINITY_DN18328_c0_g1 | AAK61826.1 | Kb1* protein | 100.00 | sp\|Q718F4\|NDB43_MESMA | Peptide BmKb1 | 100.00 |
| TRINITY_DN18328_c0_g2 | AGC92780.1 | caerin-like antibacterial peptide, partial | 79.60 | sp\|Q2M591\|NDB4T_MESMA | Peptide BmKb2 | 98.00 |
| TRINITY_DN18368_c3_g1 | XP_023210978.1 | U8-theraphotoxin-Hhn1e-like | 43.60 |  |  |  |
| TRINITY_DN1863_c0_g1 | A0F0C2.2 | Full=Mesotoxin-1 | 100.00 | sp\|A0F0C2\|SCXM1_MESMA | Mesotoxin-1 | 100.00 |
| TRINITY_DN18668_c3_g3 | Q9BJW4.1 | Full=Neurotoxin Bm12-b | 98.30 | sp\|Q9BJW4\|CTXLB_MESMA | Neurotoxin Bm12-b | 98.30 |
| TRINITY_DN18668_c3_g4 | AJT55733.1 | I4 toxin precursor | 66.00 | sp\|P86401\|CTXL_MESEU | Neurotoxin MeuClTx (Fragment) | 82.90 |
| TRINITY_DN18780_c0_g1 | ACJ23158.1 | putative toxin Tx277 | 80.00 |  |  |  |
| TRINITY_DN19065_c0_g1 | AMX81479.1 | venom peptide meuPep34 | 90.00 |  |  |  |
| TRINITY_DN19326_c3_g1 | P45697.2 | Full=Alpha-like toxin BmK-M1 | 97.60 | sp\|P45697\|SCX1_MESMA | Alpha-like toxin BmK-M1 | 97.60 |
| TRINITY_DN19326_c3_g2 | Q9NJC4.1 | Full=Toxin BmKaTx17 | 98.60 | sp\|Q9NJC4\|SC17_MESMA | Toxin BmKaTx17 (Fragment) | 98.60 |
| TRINITY_DN19326_c3_g4 | P0DMH9.1 | Full=Alpha-toxin BmalphaTx47 | 100.00 | sp\|P0DMH9\|SCX47_MESMA | Alpha-toxin BmalphaTx47 | 100.00 |
| TRINITY_DN19345_c2_g3 | ABR20114.1 | venom toxin-like peptide | 93.50 |  |  |  |
| TRINITY_DN19403_c2_g3 | XP_023217929.1 | cysteine-rich venom protein-like | 34.10 | sp\|P81656\|VA5_POLDO | Venom allergen 5 | 31.40 |
| TRINITY_DN19443_c3_g1 | AMX81473.1 | venom peptide meuPep26 | 51.70 |  |  |  |
| TRINITY_DN19489_c2_g5 | B7SNV8.1 | Full=Toxin Pg8 | 49.40 | sp\|B7SNV8\|SCX8_PARGR | Toxin Pg8 | 49.40 |
| TRINITY_DN19494_c1_g3 | P86100.2 | Full=Hyaluronidase-1 | 99.50 | sp\|P86100\|HYAL1_MESMA | Hyaluronidase-1 | 99.50 |
| TRINITY_DN19595_c1_g2 | Q86M31.1 | Full=Neurotoxin BmKAEP2 | 71.80 | sp\|Q86M31\|AEP2_MESMA | Neurotoxin BmKAEP2 | 71.80 |
| TRINITY_DN1964_c0_g1 | XP_023216994.1 | venom protein 302-like | 52.90 | sp\|P0CJ14\|VP302_LYCMC | Venom protein 302 | 51.90 |
| TRINITY_DN19792_c1_g3 | AMX81488.1 | venom toxin meuTx18 | 81.60 |  |  |  |
| TRINITY_DN1982_c0_g1 | ABR21045.1 | venom toxin-like peptide-5 | 79.20 |  |  |  |
| TRINITY_DN19846_c0_g1 | Q9GUA7.1 | Full=Toxin BmKa3 | 100.00 | sp\|Q9GUA7\|SCA3_MESMA | Toxin BmKa3 | 100.00 |
| TRINITY_DN19901_c0_g7 | Q7Z0H4.1 | Full=Neurotoxin BmP08 | 74.50 | sp\|Q7Z0H4\|SCKI_MESMA | Neurotoxin BmP08 | 74.50 |
| TRINITY_DN19987_c1_g1 | KFM77036.1 | Ras-related C3 botulinum toxin substrate 1, partial | 99.00 | sp\|P62998\|RAC1_BOVIN | Ras-related C3 botulinum toxin substrate 1 | 90.60 |
| TRINITY_DN20063_c1_g5 | BAK64109.1 | complement component 3-1 | 44.30 | sp\|Q0ZZJ6\|VCO31_AUSSU | A.superbus venom factor 1 | 27.90 |
| TRINITY_DN20087_c1_g3 | Q4TUA4.1 | Full=Alpha-toxin 4 | 81.00 | sp\|Q9GUA7\|SCA3_MESMA | Toxin BmKa3 | 81.00 |
| TRINITY_DN20215_c0_g1 | AAV64255.1 | neurotoxin Tx11 | 100.00 | sp\|Q9NJC7\|SC11_MESMA | BmK AGP-SYPU2 | 96.50 |
| TRINITY_DN20340_c6_g1 | ADT89762.1 | antimicrobial peptide marcin-18 | 100.00 | sp\|E4VP50\|NDBW_MESEU | Venom antimicrobial peptide-9 | 87.30 |
| TRINITY_DN20355_c0_g1 | AAA69557.1 | pre-Bmk1 neurotoxin | 80.90 | sp\|P45697\|SCX1_MESMA | Alpha-like toxin BmK-M1 | 80.90 |
| TRINITY_DN20355_c0_g4 | Q9NJC5.1 | Full=Toxin BmKaTx10 | 89.30 | sp\|Q9NJC5\|SC10_MESMA | Toxin BmKaTx10 | 89.30 |
| TRINITY_DN20408_c1_g1 | Q9Y0X6.2 | Full=BmK-YA precursor | 94.90 | sp\|Q9Y0X6\|YA_MESMA | BmK-YA precursor | 94.90 |
| TRINITY_DN20433_c1_g6 | PRD23776.1 | Venom peptide SjAPI-2 | 50.80 |  |  |  |
| TRINITY_DN20443_c1_g1 | Q6WJF5.3 | Full=Lipolysis-activating peptide 1-alpha chain | 90.60 | sp\|Q6WJF5\|LV1A_MESMA | Lipolysis-activating peptide 1-alpha chain | 90.60 |
| TRINITY_DN20477_c1_g2 | XP_023223251.1 | venom protein 302-like | 68.00 |  |  |  |
| TRINITY_DN20551_c0_g1 | Q7Z0F1.1 | Full=Neurotoxin X-29S | 100.00 | sp\|Q7Z0F1\|SCKH_MESMA | Neurotoxin X-29S | 100.00 |
| TRINITY_DN20564_c3_g1 | Q26292.1 | Full=Beta-insect depressant toxin LqhIT2 | 60.50 | sp\|Q26292\|SIX2_LEIQH | Beta-insect depressant toxin LqhIT2 | 60.50 |
| TRINITY_DN20584_c0_g2 | P0DMH9.1 | Full=Alpha-toxin BmalphaTx47 | 100.00 | sp\|P0DMH9\|SCX47_MESMA | Alpha-toxin BmalphaTx47 | 100.00 |
| TRINITY_DN20590_c2_g2 | AIX87701.1 | orphan peptide AbOp-14 | 80.00 | sp\|C6ZH25\|VP1_LYCMC | Venom protein TxLP11 | 38.40 |
| TRINITY_DN21108_c0_g1 | ABR21044.1 | venom insulin-like growth factor binding protein-1 | 92.10 | sp\|Q61581\|IBP7_MOUSE | Insulin-like growth factor-binding protein 7 | 42.10 |
| TRINITY_DN21163_c0_g1 | XP_023210205.1 | venom protease-like | 83.60 | sp\|P21902\|PCE_TACTR | Proclotting enzyme | 43.50 |
| TRINITY_DN21211_c0_g1 | XP_023215406.1 | venom protein 30.1-like | 77.20 |  |  |  |
| TRINITY_DN2266_c0_g1 | AAK61817.1 | putative anticoagulant peptide AP1 | 98.80 | sp\|Q86RQ7\|VPI_MESMA | Venom peptide BmKAPI | 97.60 |
| TRINITY_DN2281_c0_g1 | XP_023230486.1 | U8-agatoxin-Ao1a-like | 92.40 |  |  |  |
| TRINITY_DN2419_c0_g1 | ALX72368.1 | venom protein VP3 | 85.80 |  |  |  |
| TRINITY_DN2657_c0_g1 | D2CFI7.1 | Full=Venom peptide MmKTx1 | 100.00 | sp\|D2CFI7\|LA1_MESMA | Venom peptide MmKTx1 | 100.00 |
| TRINITY_DN3323_c0_g1 | XP_023964654.1 | fused toxin protein | 54.10 | sp\|P00993\|IBP_CARCR | Chelonianin | 52.60 |
| TRINITY_DN3440_c0_g1 | XP_023227203.1 | venom protein 29-like | 46.80 |  |  |  |
| TRINITY_DN3616_c0_g1 | XP_023216994.1 | venom protein 302-like | 63.70 | sp\|P0CJ14\|VP302_LYCMC | Venom protein 302 | 44.20 |
| TRINITY_DN3890_c0_g1 | Q5F1N4.1 | Full=Toxin BmTxKS4 | 98.70 | sp\|Q5F1N4\|KS4_MESMA | Toxin BmTxKS4 | 98.70 |
| TRINITY_DN451_c0_g1 | XP_023211714.1 | U24-ctenitoxin-Pn1a-like | 74.20 | sp\|P31226\|SAX_LITCT | Saxiphilin | 46.90 |
| TRINITY_DN4705_c0_g2 | XP_023212786.1 | venom allergen 5-like | 75.50 | sp\|B2MVK7\|VA5_RHYBR | Venom allergen 5 | 40.90 |
| TRINITY_DN4733_c0_g1 | AMX81486.1 | venom toxin meuVNP2 | 98.80 |  |  |  |
| TRINITY_DN5031_c0_g1 | AHZ63123.1 | putative venom peptide | 96.10 |  |  |  |
| TRINITY_DN6041_c0_g1 | XP_023239555.1 | cysteine-rich venom protein-like | 67.30 | sp\|Q8AVA4\|CRVP_PSEAU | Cysteine-rich venom protein pseudechetoxin | 30.40 |
| TRINITY_DN6105_c0_g1 | ABR21061.1 | venom protein-7 | 97.80 |  |  |  |
|  |  |  |  | sp\|Q9FFP8\|DEF06_ARATH | Defensin-like protein 6 | 56.20 |
|  |  |  |  | sp\|P81009\|DEF2_MAIZE | Defensin-like protein 2 | 100.00 |
| TRINITY_DN6646_c0_g1 | XP_023241610.1 | toxin-like protein 14 | 55.70 |  |  |  |
| TRINITY_DN7881_c0_g1 | XP_023234358.1 | venom protein 302-like | 59.30 | sp\|P0CJ14\|VP302_LYCMC | Venom protein 302 | 46.60 |
| TRINITY_DN8648_c0_g1 | XP_023217417.1 | venom protein 302-like | 51.30 |  |  |  |
| TRINITY_DN934_c0_g1 | AAK61818.1 | putative toxin-like peptide KTXLP2 | 80.00 | sp\|Q95P90\|LV1B_MESMA | HMG-CoA reductase inhibitor bumarsin | 79.80 |
| TRINITY_DN9851_c0_g1 | P0CJ18.1 | Full=Venom protein 30.1 | 54.10 | sp\|P0CJ18\|VP30_LYCMC | Venom protein 30.1 | 54.10 |
| TRINITY_DN9851_c0_g2 | XP_023220611.1 | venom peptide MmKTx1-like | 59.30 |  |  |  |
| TRINITY_DN991_c0_g1 | XP_023238900.1 | toxin KTx8-like | 70.80 | sp\|A9QLM3\|KA11L_LYCMC | Toxin KTx8 | 69.20 |
